# Spatial Distribution of Tropospheric Ozone in National Parks of California: Interpretation of Passive-Sampler Data

**DOI:** 10.1100/tsw.2001.83

**Published:** 2001-09-28

**Authors:** John D. Ray

**Affiliations:** National Park Service, Air Resources Division, 12795 W. Alameda Parkway, P.O. Box 25287, Denver, CO 80225-0287, USA

**Keywords:** air pollution, national parks, passive samplers, kriging, spatial interpolation

## Abstract

The National Park Service (NPS) has tested and used passive ozone samplers for several years to get baseline values for parks and to determine the spatial variability within parks. Experience has shown that the Ogawa passive samplers can provide ±10% accuracy when used with a quality assurance program consisting of blanks, duplicates, collocated instrumentation, and a standard operating procedure that carefully guides site operators. Although the passive device does not meet EPA criteria as a certified method (mainly, that hourly values be measured), it does provide seasonal summed values of ozone. The seasonal ozone concentrations from the passive devices can be compared to other monitoring to determine baseline values, trends, and spatial variations. This point is illustrated with some kriged interpolation maps of ozone statistics. Passive ozone samplers were used to get elevational gradients and spatial distributions of ozone within a park. This was done in varying degrees at Mount Rainier, Olympic, Sequoia–Kings Canyon, Yosemite, Joshua Tree, Rocky Mountain, and Great Smoky Mountains national parks. The ozone has been found to vary by factors of 2 and 3 within a park when average ozone is compared between locations. Specific examples of the spatial distributions of ozone in three parks within California are given using interpolation maps. Positive aspects and limitations of the passive sampling approach are presented.

### INTRODUCTION

An extensive network of ozone monitoring stations exists in the U.S.; however, most of these monitors are located in urbanized areas. Because most of the precursor emissions and the highest ozone values are generally found in urban areas, a higher density of monitoring stations has been used to characterize the spatial patterns of ozone among the population centers. In recent years it has become evident that rural national parks, including several in California, have ozone concentrations[1] that exceed the National Ambient Air Quality Standard (NAAQS) that was designed to protect human health and welfare (which includes natural resources). A limited number of ozone monitors have been used to observe what is thought to be primarily transported ozone. Because only limited emissions are local to these monitors, the assumption has been that the measurements are representative of ozone concentrations over areas anywhere from 1 to 60 km or more from the station. Three national parks within California provide rural areas with significant natural resources where complex terrain and vegetation patterns make it unlikely that the ozone distribution is uniform.

This study uses passive ozone samplers that are inexpensive and do not require a power source to make direct measurements within the geographic areas of interest. Passive samplers based on the reaction of ozone with nitrite have been reported to be selective for ozone, linear in response, and relatively unaffected by environmental changes such as temperature, humidity, solar radiation, and pressure[2,3,4,5]. Some sensitivity to other pollutants has been reported[6] at high concentrations that would be found only near major sources or in major cities. The passive samplers have been used to characterize the ozone distribution around urban areas in Toronto, Canada[7]; Dallas–Fort Worth, Texas[8]; and Mexico City, Mexico[9]. When 12- or 24-h averages were used in the Dallas–Fort Worth study[8], ozone was found to vary by 5 to 10 ppb over distances of 20 km at monitoring sites 60 km from an urban center. High ozone concentrations that exceeded the 8-h NAAQS were found by passive samplers at distances greater that 120 km from the central city. A network of passive monitors had a correlation coefficient between samplers that decreased linearly with distance to about 150 km[8].

Interpolation of ozone data at western rural locations has been reported by Lee[10] using state and federal ozone monitoring data. The detailed maps in Lee’s publication might be compared to the ozone contour maps presented here. Results from joint projects with the National Park Service (NPS) and researchers from the University of Washington have been presented as interpolated ozone maps for Mount Rainier[11] and other parks in Washington[12]. Peterson[13] and Cooper and Peterson[14] have used passive samplers in the consideration of spatial and temporal scale for the placement of monitoring stations.

The expected ozone concentration is a function of the scale being considered. In this paper, distances of a few meters to hundreds of meters are considered. Interpolation and mapping methods are used to estimate ozone concentrations and the gross transport pathways used to explain the observed patterns. The capabilities and limitations of the passive samplers are examined and compared to the information provided by the hourly-data ozone monitors.

### EXPERIMENTAL METHODS AND PROCEDURES

A rural monitoring network of ozone analyzers is operated in the western U.S. by the NPS to determine base-line conditions and trends. These monitoring stations meet EPA guidelines for data collection and include a full suite of meteorological sensors. A secondary network of passive sampler monitoring is conducted routinely in 18 park units (Fig. 1) and some passive sampler monitoring has been done in 28 parks, 10 with multiple samplers.

Network operations for the passive program are generally between May and the end of September each year. Spatial distribution studies with multiple sampling locations within a park are more intensive and are limited to periods ranging from three weeks to a 5-month season. The sampling locations are selected with the help of field staff based on geographic distribution, accessibility, experimental design, elevation, and security. Data from the two networks are compared and combined to determine baseline conditions and trends.

Several different portable ozone analyzers and passive sampler methods were evaluated in field trials. The Ogawa (http://www.ogawausa.com) passive samplers were selected after trials in

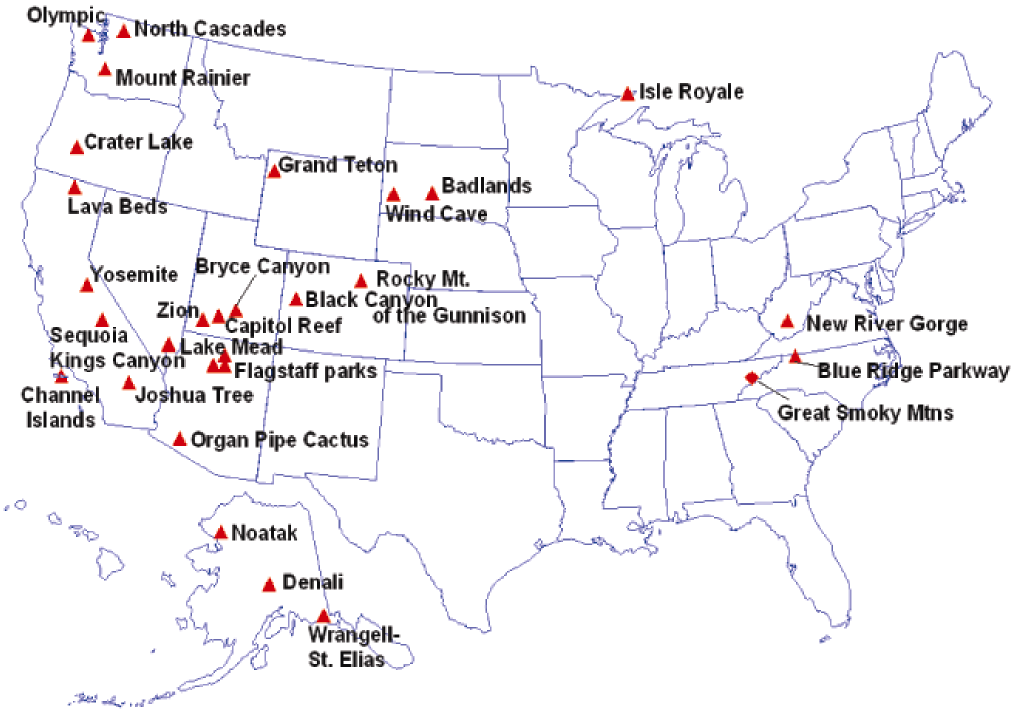

**FIGURE 1**. National park locations where passive ozone monitoring have been used. Eighteen of the parks have no other ozone monitoring and the passive sampling is being used to determine baselines and trends.

a broad cross section of environments and geographical locations for use in NPS units. Ogawa samplers consist of a double-sided filter holder that is mounted on a badge with a clip on the back. Inside the filter holder are two nitrite-coated filters. Airflow into the sampler depends solely on diffusion, i.e., there are no active components in the sampler to draw air in. When the nitrite-coated filters are exposed to ambient air, ozone diffuses through the end-caps and reacts with the nitrite to form nitrate. A rainshield of PVC plastic protects the samplers from direct contact with water and sunlight in field use (see Fig. 2A) to avoid artifacts or low readings. The on-site equipment consists of a 3.5-m PVC plastic-pipe “tower” and a pair of PVC rainshields that remain at the sampling site (Fig. 2B). The passive samplers are mounted on badges that clip to the support inside the rainshields. Standardization of the field equipment and all the procedures has been a big factor in achieving consistent results.

### Passive Samplers and Design of Equipment

The passive samplers are shipped to the field sites inside amber plastic vials and zip-closure plastic bags. Color-coded plastic tape is used to make sure pressure changes during shipment do not pop the lids off and to identify the functions of the samplers. When field personnel remove the sampler from the protective containers, exposure timing begins. After a measured time of exposure (normally 1 week, though periods of up to 1 month have been used successfully), the samplers are returned to the shipping containers and return-shipped to the lab for analysis. At the lab, the filters are removed in an ozone-free atmosphere, the filters extracted with water, and the extract analyzed by ion chromatography for nitrate ion. The mass of the nitrate is used to calculate[2,13] the ozone dose or the average ozone hourly concentration which is determined by dividing ozone dose by the exposure time.

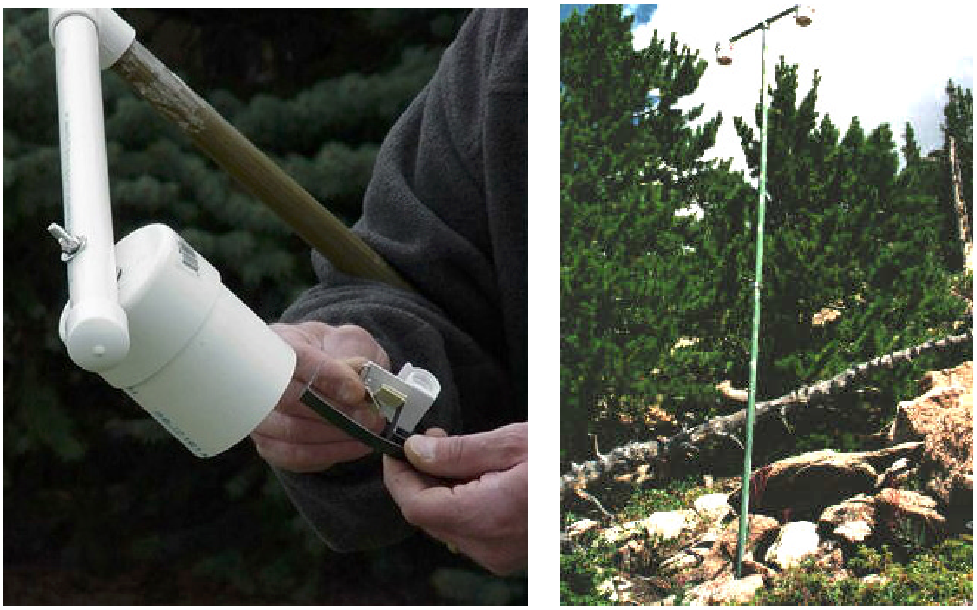

**FIGURE 2**. Pictures of the field equipment used with the passive sampler badges. *Left:* Rainshield and clip to hold badges. *Right:* sampling system pole with two rainshields in field use. The sampling systems are placed in clearings such as the rock field shown. The poles and the outside of the rainshields are painted to blend into the surroundings.

### Calculations, Data Handling, and Analysis

Passive samplers are shipped to the park once a month. Each shipment contains enough samplers for each exposure period during the month plus "blanks" that are not exposed, and some samplers for duplicate exposures. Each plastic vial containing a passive sampler has a label for the date and times to be recorded. In addition, the dates, times, sample numbers, and local observations are recorded on a log sheet. At the end of the month, park personnel return the exposed samplers and associated blanks in the shipping box to the lab using a prepaid shipping label. The lab reports nitrate mass and the blank values. The exposure times are then used to calculate an average weekly ozone concentration in ppb.

Quality control is important to get good results from network and intensive passive sampler studies. Sample handling, coding, and shipping are designed and controlled to minimize mishandling by the part-time field operators and misunderstandings by the lab staff. The blanks and duplicates are checks on the sampling and handling. Several locations are chosen each season for collocated measurements with a continuous ozone analyzer so that the accuracy and collection factor can be checked. All spatial distribution studies have one or more collocated measurement sites. Data validation screening is the final step before calculated ozone concentrations are released. Training materials, standard operating procedures, log sheets, and results are posted to a Web site at http://www2.nature.nps.gov/ard/gas/passives.htm.

### Locations and Experimental Design

The ozone spatial distribution studies in this report are California and western U.S. sites in national parks as shown in Fig. 1. The Joshua Tree National Park (NP) 1997 study included six passive sampler sites and one collocated station in the western half of the park. Data were obtained from the EPA's Aerometric Information Retrieval System database for several continuous ozone monitors that are outside the park, and included in the analysis. At Sequoia–

Kings Canyon NP, seven passive ozone sampler sites, three continuous monitors, and a collocated site were used in a 1998 study. Sampling at Yosemite NP was at 11 passive ozone sites and 1 collocated continuous ozone-monitoring site. The Yosemite study was designed as transects rather than a grid of measurements. In each case, the objective was to increase the level of detail on the distribution of ozone over these geographically diverse areas.

Site locations were selected that had clearings of 20 m or larger. Prior studies had shown equivalence between 10 m and 3.5 m tower-based measurements within the precision of the passive samplers. No difference was observed between the center of a clearing and the edge; however, ozone values within the forest were found to be slightly lower than in a clearing. Measurements in several different forest types have shown ozone gradients within the forest to be highest at the top of the canopy. A regular grid of sites produces the best interpolation results, but grid subsets can work if they are distributed throughout the domain of interest. The studies reported here are a balance between the ideal grid placement in open areas and the cost, manpower, and constraints of site access.

## RESULTS AND DISCUSSION

### Accuracy, Precision, and Linearity

The reliability of the passive samples under field conditions was checked carefully in preliminary studies in different ecosystems distributed over the continental U.S. The passive sampler collection factor varied by a small amount at each location where collocated passive and continuous monitors were located. Because ozone monitors operated according to EPA guidelines are typically within ±5% but can be as high as ±15%, the passive samplers would be expected be vary by at least a similar amount. Fig. 3 shows the comparison of passive and continuous monitoring data at collocated sites in seven western parks. The linear regression shows a nearly 1:1 relationship when an empirically determined average collection factor over all parks of 21.656 cm is used in the ozone calculation. The ±5 ppb concentrations about the regression line contain most of the data points in the collocated monitoring data in Fig. 3. Early lab work reported a substantially different value for the collection factor[2,13]; however, other field studies[3,9] have used a similar value.

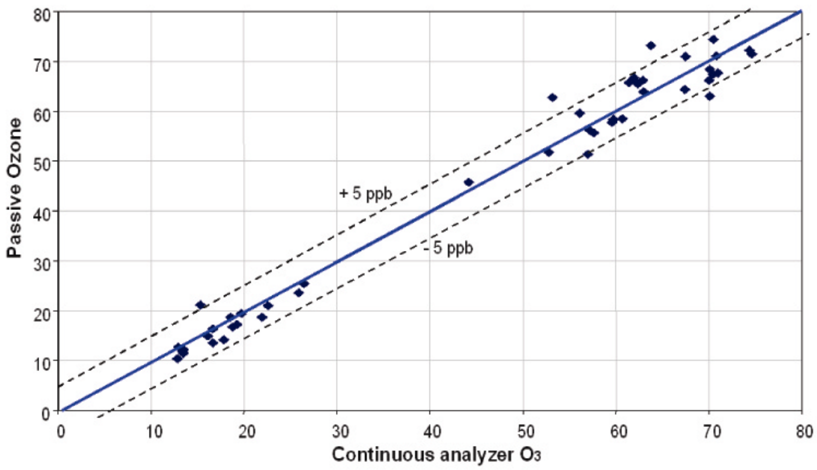

**FIGURE 3.** Comparison of the passive ozone to collocated EPA-certified continuous ozone monitors operated according to EPA guidelines and calibrated with transfer standards. The solid line shows the 1:1 relationship to assist in visual comparison.

**TABLE 1**
**Precision and Accuracy for the Passive Samplers**

| | Precision | | | Accuracy | |
|---|---|---|---|---|---|
| Year | n | Pooled Std Dev | % RSD | n | Mean Absolute % Diff. |
| 1995 | 218 | 1.36 | 2.42 | 5 | <10 |
| 1996 | 202 | 2.05 | 2.04 | 39 | 7.7 |

*Note*: Based on collocated sites over a two summer sampling periods. RSD = relative standard deviation; units for standard deviation are ppbv.

If the individual monitoring sites were broken out they would cluster in different concentration ranges along the line; for example Olympic NP values are clustered in the 5 to 20 ppb range, but have a similar distribution at each site. The best indicator of the variability or precision in the measurements is from a paired standard deviation from the duplicate samples. Table 1 gives the results from two different years for more than 400 passive sampler measurements. For weekly exposures, the standard deviation in the ozone concentrations is 1.4 to 2.1 ppb. The difference between years related more to absolute value of ozone measured as indicated by the relative standard deviation percent (RSD %), which is around 2% for each year. The mean absolute percent difference between the passives and the collocated continuous monitor data was less that 10%. The precision results scale proportionally if shorter exposure times are used.

The key conclusion about the passive ozone samplers is that over a variety of monitoring locations, elevations, ecosystems, and weather conditions in North America, the expected ozone results are linear and within 10% of the value that would be obtained from an EPA certified ozone analyzer. No systematic bias is observed with different ranges of ozone, elevation, temperature, or relative humidity. There is small variability between locations in the observed collection factor that has not been explained, but these variations do not cause the ozone values to be in error by more than ±10%. The cause of the variability by site may be that the collocated continuous analyzers are monitoring instruments and not calibration devices. Rather than correct the collection factor for each location based on a collocated analyzer, which itself may be inaccurate, an average factor is used for all the calculations and checked each year to ensure nothing has changed in the devices.

Results from the passive sampler studies are used here to examine the distribution of ozone in the western U.S. on different scales. The first scale is in the hundreds of km range, which encompasses the whole of the western U.S. Average ozone concentrations from both continuous monitors and passive samplers for the season May to September are presented for 1997 in Fig. 4.

These data represent the rural ozone concentrations and are a good indication of the current continental background ozone concentrations. The lowest ozone concentrations are in the northwest and across the northern states of the U.S. The highest ozone concentrations are centered around California, which is consistent with pollutant transport from major polluted urban centers. The passive ozone data fills in almost half of the locations.

The passive samplers pick up week-to-week tendencies that relate to weather changes just as the continuous analyzers do. For each season a distribution of weekly ozone values is obtained, which has been compared to the ozone distributions from collocated continuous analyzers. The similarity of the distribution leads to the conclusion that an estimate of the maximum hourly ozone for the season can be estimated from the data. A linear relationship between the maximum seasonal ozone and the average $O_3$, as determined by the passive ozone sampling, has been

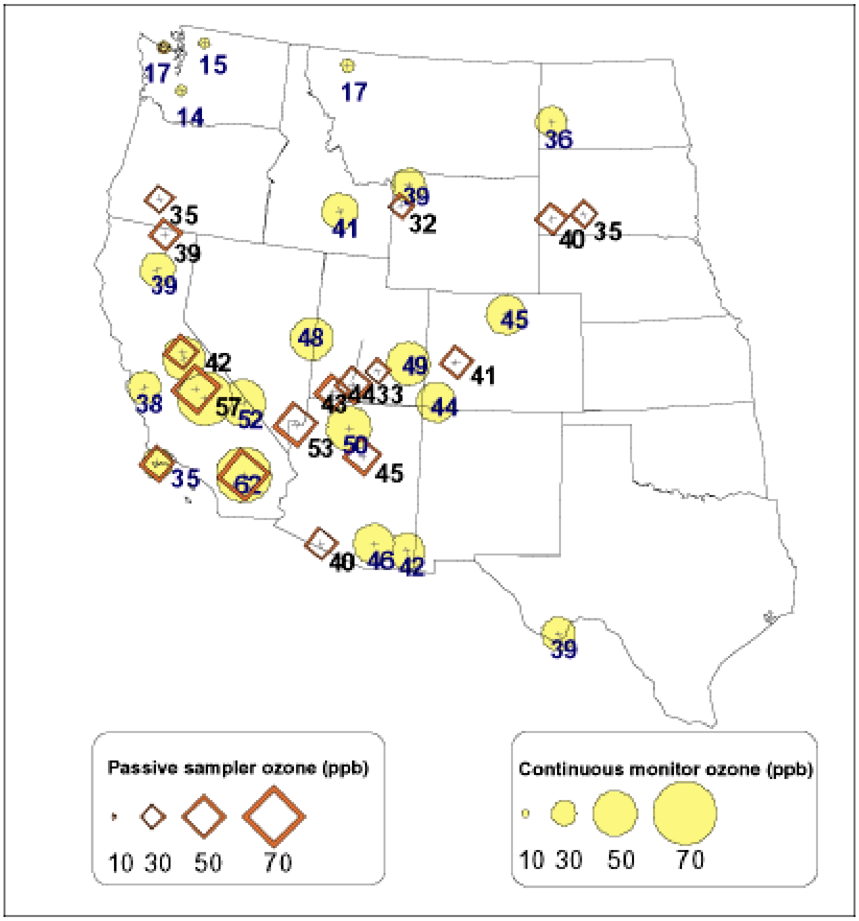

**FIGURE 4.** Average 1997 seasonal ozone concentrations based on NPS measurements at rural western U.S. park locations. The size of the symbols is proportional to the ozone concentration. Passive sampling sites are marked by diamonds, and continuous analyzer sites are marked by filled circles.

developed using the passive and collocated continuous sampler data (Seasonal max. $O_3$ = 1.18 * (avg. $O_3$) + 20.3 $R^2$ = 0.89). The uncertainty in the maximum seasonal 1-h ozone is approximately ±6 ppb. This approximation method is far from perfect, but allows an estimation of the seasonal high ozone concentrations for a site.

The point measurement presentations in Fig. 4 are strictly correct from a presentation point of view, but what if an estimate of the ozone is desired at some place other than the exact monitoring location? One approach is to take the value of the nearest measurement. But the western U.S. is known for its high mountains, deep canyons, forests, and deserts. An alternative approach is to do linear interpolations between multiple monitoring stations. Fig. 5 shows the result if the kriging technique is used with the data in Fig. 4. These estimates of ozone concentrations begin to hint at areas that are major source regions and how transport patterns may affect the background ozone. Resolution may be the real issue here. With the spacing of measurement points, topographical features that are 50 to 100 km wide that would surely affect ozone concentrations are simply not seen. For example, can this map represent the ozone at the bottom of the Grand Canyon or the eastern edge of the Sierra Nevada Mountains, or the difference between the eastern and western divide in Rocky Mountain NP? Direct measurements with passive samplers at Rocky Mountain NP showed a factor of three difference in ozone on the east and west sides of the divide. Of course, urban source areas that have high ozone — Salt Lake City or Los Angeles, for example — do not appear.

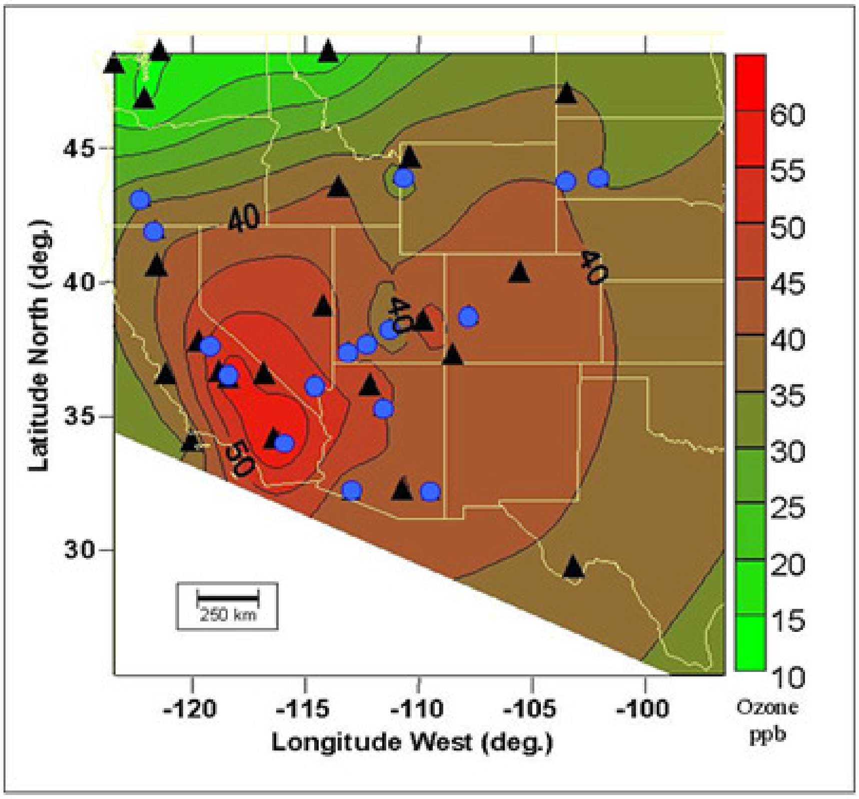

**FIGURE 5.** Rural ozone concentrations as seasonal averages over the western United States in 1997 based on measurements of passive and active monitors at the national parks marked. Black triangles are continuous analyzer sites and the blue dots are passive sampler location.

The main value of the map in Fig. 5 is that it shows connections and promotes questions. A broad area of California is estimated to have high ozone and just happens to be downwind of some major urban areas that are known to have high ozone.

Based on this interpolation map, most of the southern Sierra Nevada mountains are projected to have high ozone. A broad central area of the western states appears to have elevated background ozone based on this projection. Large areas of Nevada, Utah, and Colorado are sparsely populated and do not have large amounts of precursor emissions. The measured ozone values appear to be higher than what would be expected; transport of ozone and precursors over hundreds of km is suggested.

Direct ozone measurements with multiple continuous analyzers in Sequoia–Kings Canyon and Yosemite national parks have shown that the concentrations throughout the mountains are not uniform. Variations in ozone occur in the 10- to 50-km range. To get that kind of resolution, a much denser network of monitoring stations is required. But remote mountainous regions of the parks do not have electrical power, and the hundreds of monitors would be prohibitively expensive. Some short-term passive ozone monitoring networks have been used to get the spatial distributions at this scale. Three examples of ozone interpolation in California are given here.

### Ozone in Joshua Tree National Park

Joshua Tree National Park was used for one trial of the passive ozone samplers because it has high ozone concentrations that routinely exceed the recent one-hour national standard and the current eight-hour national standard. The continuous ozone analyzer for the park is located at Black Rock in the northwest corner of the park in San Bernardino County; however, most of the park is located south and east in Riverside County. Mountains define the edges of the park, especially on the south, and high desert areas are in the interior. Los Angeles is to the west and separated from the park by the San Bernardino and Santa Rosa mountains and Banning Pass. The broad Coachella Valley lies to the south, and the high desert area of the Mojave lies to the north. A prior monitoring station had been located in the central desert of the park near Lost Horse, which had also shown high ozone and many exceedances of the national ozone standard. Six passive samples were positioned around the western half of the park to determine the distribution of ozone during a June to September period when the highest ozone values were expected. Weekly samples in duplicate were taken and the collection factor was checked by a collocated passive sampler and a continuous analyzer at the Black Rock monitor.

A comparison of the Joshua Tree NP, Black Rock station maximum seasonal ozone concentrations to other nearby monitoring stations shows that the values correspond with a decreasing trend going eastward. Table 2 shows maximum ozone concentrations for the period along two rough transects going west–east and north–south. The west–east gradient in ozone is quite pronounced. The north–south transect is less regular. If the center of Los Angeles is taken as point zero and distances to the monitoring sites are considered, there is a linear decrease in maximum ozone along the west–east transect. The results of the interpolation analysis, using the kriging technique, are shown in Fig. 6. Data from surrounding continuous monitors have been included to better define the edges of the domain. The ozone interpolation map (Fig. 6) indicates some important features for the area.

The western park area and surrounding Coachella Valley and San Bernardino County north of the park have similar ozone concentrations. The ozone concentrations tend to decrease to the east and south. The highest ozone concentrations for the weekly averages were at the highest measurement site at Keys View in the Little San Bernardino Mountains. An ozone gradient with elevation is implied from the figure and the data. When ozone data from the west in the Los Angeles basin were averaged over the same weekly and seasonal periods, the concentrations were actually lower than for the park and Coachella Valley. Two factors could account for this: overnight titration of ozone by the greater NO emissions in the urbanized area and transport of precusors to the east with ozone formation occurring east of the Los Angeles basin. The time of day for the maximum ozone concentrations at Black Rock is between 5 and 7 p.m. Given that the high temperature and sunlight for the park area occurs 4 to 5 h earlier, transport of ozone and precursors would appear to be an important factor. The coarser ozone map in Fig. 5 implies much the same thing, but with less detail for the area around the park.

**TABLE 2**
**Comparison of Maximum Seasonal Ozone at Other Monitoring Stations in 1998**

| **Transect** | **Glendora** | **Riverside** | **Banning** | **Palm Springs** | **Joshua Tree** | **Indio** |
|---|---|---|---|---|---|---|
| West–East (ppb) | 222 | 193 | 169 | 173 | 142 | 134 |
| Distance from LA (km) | 1 | 69 | 114 | 156 | 172 | 207 |
| **Transect** | **Trona** | **Barstow** | **Victorville** | **Hesperia** | **Joshua Tree** | **Palm Springs** |
| North–South | 109 | 100 | 162 | 159 | 142 | 173 ppb |

*Note*: units are ppb as 1-h daily maximum values.

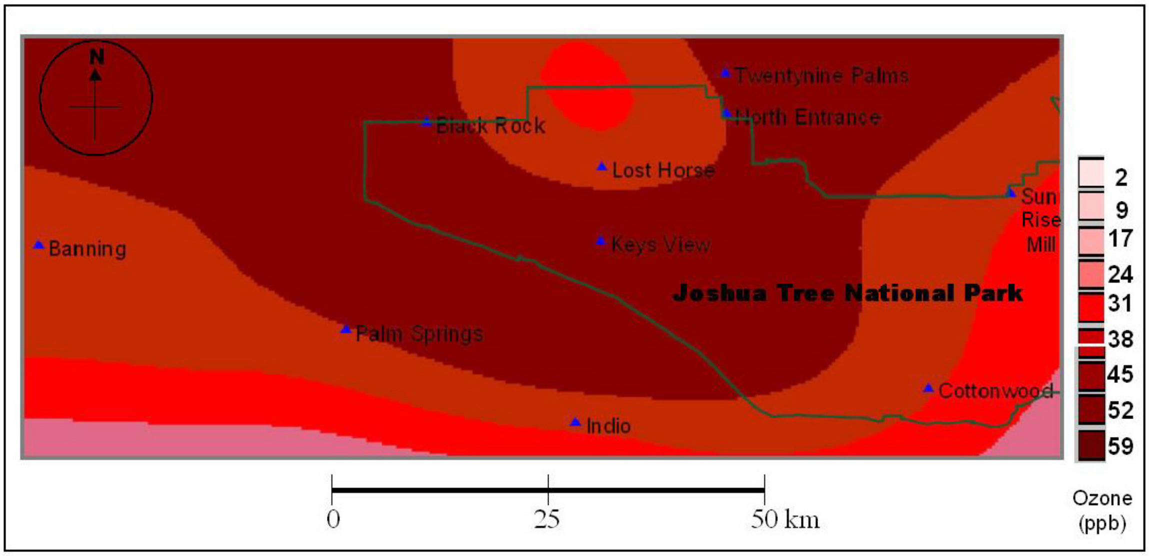

**FIGURE 6.** Spatial distribution map of kriging interpolated seasonal average ozone for Joshua Tree NP, California. The approximate boundary for the park is shown.

### Ozone in Sequoia–Kings Canyon National Park

A spatial distribution study at Sequoia–Kings Canyon NP used seven passive samplers and three continuous ozone analyzers. The park is situated to the east of the San Joaquin Valley in the Sierra Nevada mountain range. The cities of Fresno and Visalia are to the west in the valley. The roads and major access to these adjoining parks are along the western edges where the continuous ozone monitoring has traditionally been because power and access were available.

The passive samplers provided a means to estimate ozone concentrations in the interior and eastern side of the parks (Fig. 7). The interpolation map indicates that the highest ozone concentrations are in the southwestern corner of Sequoia NP. This area is the headwaters for the Lower Kaweah river drainage that provides a route for polluted San Joaquin air to rise out of the valley during the daytime heating. A distinct up-slope, down-slope diurnal wind pattern is observed at the monitoring sites. At the Giant Grove (Lower Kaweah) monitoring station, an inversion layer with brown hazy air is often observed in the morning in the San Joaquin Valley to the west. The inversion layer is observed to raise during the day until the hazy air obscures the view. The passive sampler sites extended the measurements up into the mountain peaks and show a decreasing gradient of ozone with elevation. Both the eastern side of the park and the northern areas have much cleaner air according to the plot in Fig. 7.

What Fig. 7 does not show are the high mountain peaks that form a barrier ridge running north–south through the park. It is very likely that the eastern side of the park is distinctly different in ozone concentrations. In this study there was not enough sample points to clearly show ozone along either the ridge or the lower elevation eastern side. A spacing of 10 to 20 km between sites could show the mountain barrier if several sites were along the high mountain ridge. A comparison of Fig. 7 and Fig. 5 shows how the more widely spaced western U.S. data gets the general features but is unable to distinguish either natural barriers or the polluted San Joaquin Valley. Thus, high ozone concentrations in Fig. 5 are projected farther to east that what actually appears to happen.

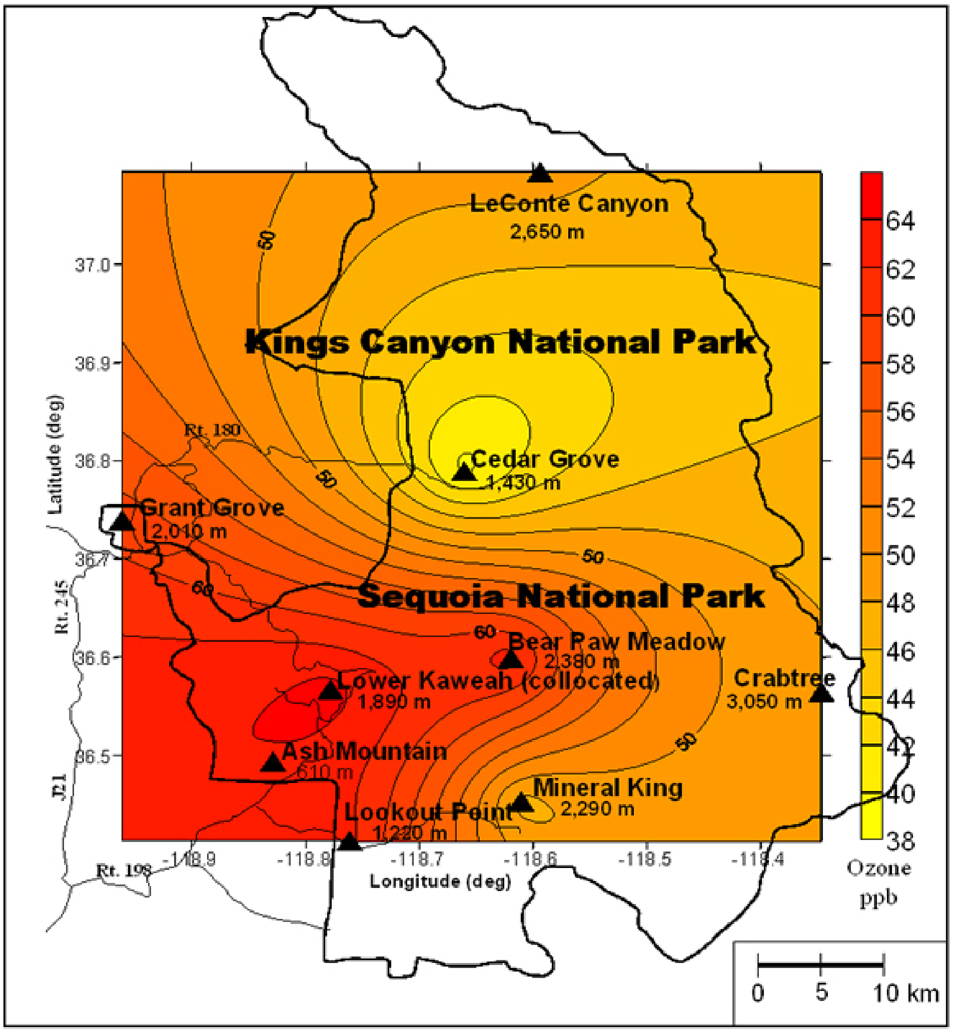

**FIGURE 7.** Spatial distribution map of kriging interpolated seasonal average ozone for Sequoia–Kings Canyon national parks.

### Ozone in Yosemite National Park

Passive ozone samplers have been used in Yosemite NP to better understand the distribution of ozone over the Sierra Nevada range and the scenic valleys. In 2000, 11 passive samplers and 1 continuous ozone monitor were used along three transects (Fig. 8). One transect was along the western edge of the park, one up Yosemite Valley, and another along the Tioga Road that crosses through the park.

The distribution of sampling points is not well suited to the kind of interpolations used in the other two studies. Also, the air transport patterns are more complex since Yosemite is separated from the San Joaquin Valley by intervening mountains and by longer, more complex drainages. The present continuous ozone monitor is at Turtleback Dome, which sits above the Yosemite Valley on the western edge. There are several reasons that this monitor might not be representative. A layer of smoke is observed to hang in the Yosemite Valley during early morning that is well below the Valley rim. This inversion layer suggests that the Valley is cut off from the monitoring station for most or all of the day.

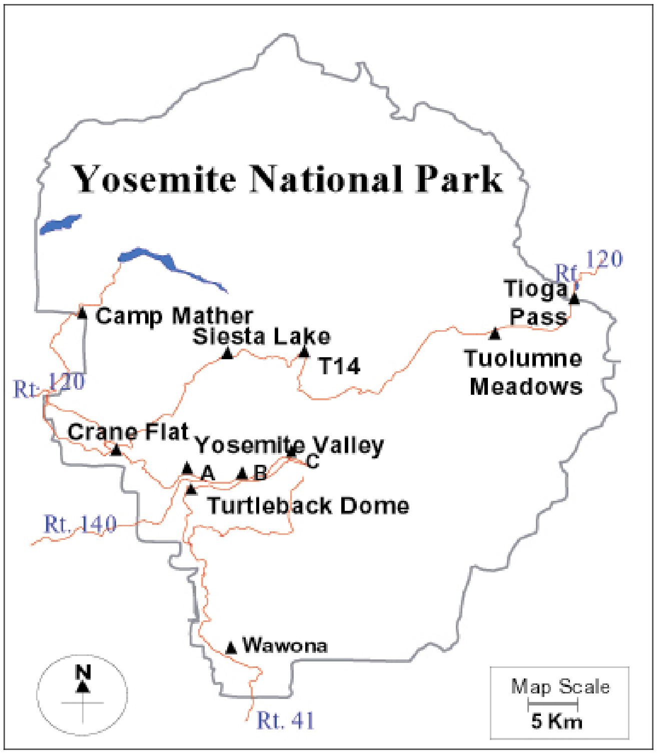

**FIGURE 8.** Locations of passive monitoring sites in Yosemite NP. Sites are accessible by a hike within 15 min of the roads. The sampling poles are placed out of direct public view.

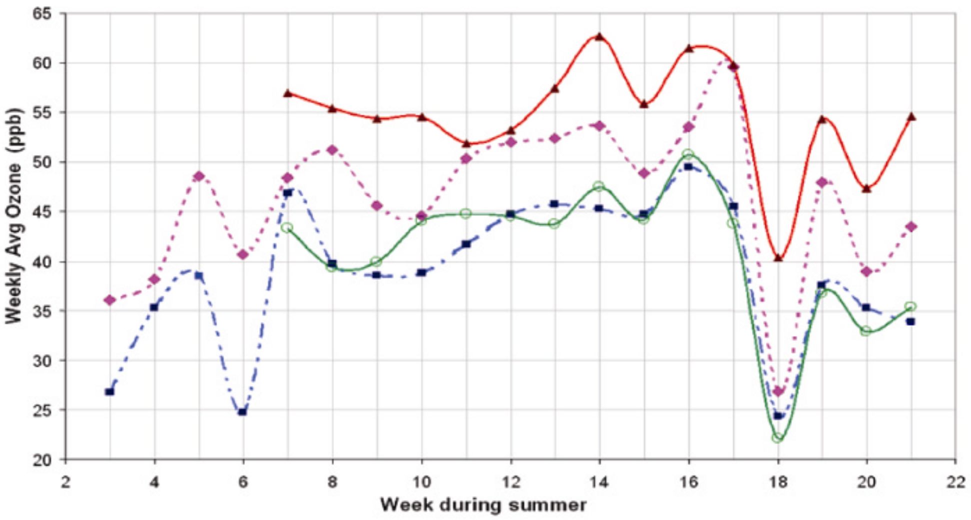

**FIGURE 9.** Comparison of weekly average ozone concentrations at three western-boundary sites and a site within Yosemite Valley. The Turtleback Dome site is on the rim and well above the valley floor. The other three sites are lower and about the same elevation. *Legend:* triangle – Turtleback Dome; diamond – Camp Mather; square – Wawona Valley; open circle – Yosemite Valley.

In Fig. 9, three monitoring sites along the western edge of the park are compared to measurements of ozone in the Yosemite Valley based on weekly averages. Although the Yosemite Valley follows the weather-related up-and-down pattern seen at Turtleback Dome, the ozone concentrations are considerably lower in the Valley.

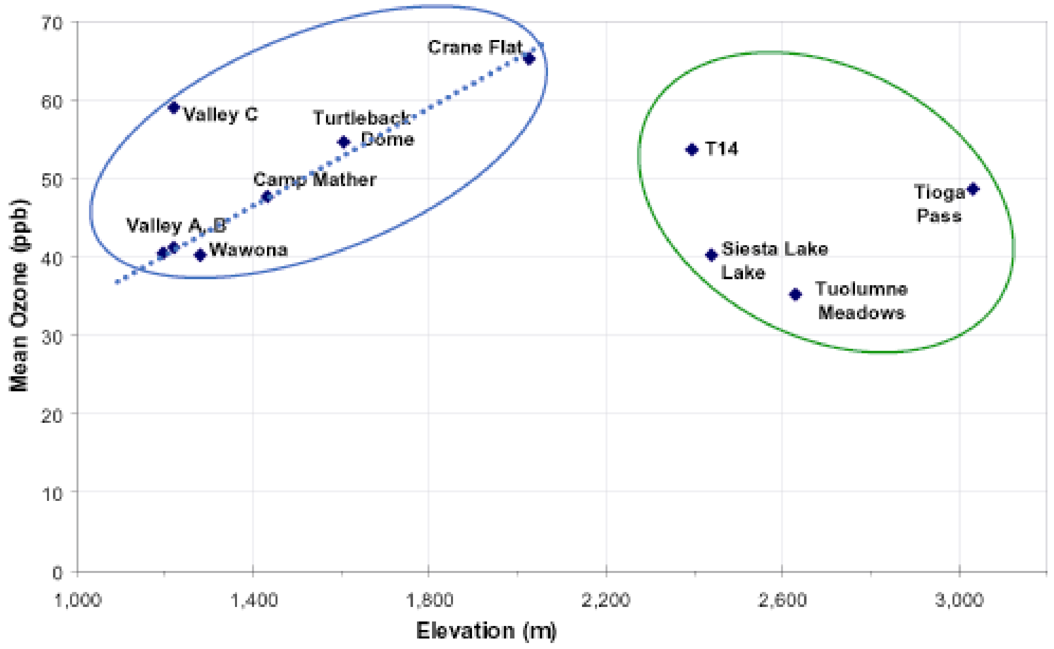

**FIGURE 10.** Mean seasonal ozone concentrations plotted against elevation for each of the passive ozone measurement sites in Yosemite NP. Factor analysis of the weekly data separates the sites into two groups. The west-side sites increase in ozone concentration with elevation up to about 2000 m.

The Camp Mather and Wawona Valley sites are intermediate in elevation between the Yosemite Valley and Turtleback Dome sites. Camp Mather has intermediate concentrations of ozone and Wawona Valley has values nearly the same as Yosemite Valley. The Yosemite Valley site can be related to the Turtleback Dome site with a linear regression equation ($R^2 = 0.75$) of slope 1.1 and intercept of –20 ppb. The bias between the sites can be explained by air being trapped in Yosemite Valley overnight that deposits ozone to surfaces and ozone reaction with NO from vehicle emissions. During the day air from outside the valley mixes in, which raises the ozone concentrations to values closer to the full-time monitor observations.

If the passive ozone data for Yosemite are plotted by elevation (Fig. 10), two patterns are seen. The sampling sites within the blue ellipse have an ozone gradient that increases with elevation up to about 2000 m. Yosemite Valley site C is at Yosemite Village where vehicle traffic and activity are high. Ignoring site C, the ozone increases at 3.1 ppb/100 m ($R^2 = 0.98$, intercept of 2.5 ppb). Higher elevation sites are variable and do not show a consistent gradient up or down. Factor analysis of the data over the whole summer season also separated the sites as in Fig. 10. One explanation could be that the sites below Crane Flat are influenced by up-slope flows that bring ozone through complex pathways from the ozone-rich San Joaquin Valley. In essence at this height and above, the sampling sites are influenced mostly by free tropospheric air that is less directly connected with a specific source or polluted area.

### CONCLUSION

The question of representativeness of a monitoring site and the variablility of ozone concentrations in a rural environment has been examined here. Within the bounds of the detection limit, cost of sampling, and the time required to get samples, a simple passive sampler device has been shown to provide weekly and seasonal average ozone concentrations with an accuracy of better than ±10%. Exploratory studies using a combination of passive and active monitoring devices gave estimates of the variability of ozone in the topographically and environmentally complex terrain of some major western national parks. In the three cases examined here, the continuous monitor was found to be in a location that represented the upper end of the observed ozone. Higher ozone concentrations within the parks were generally found at higher elevations, at locations closer to major precusror source areas, or along drainages that served as polluted-air transport corridors. The variablity in seasonal ozone for different areas within the parks studied was such that the highest ozone areas were a factor of 2 or more above the areas with lowest ozone. Thus, uniformity of ozone concentrations can not be expected within a park.

The passive ozone samplers have been shown to be useful in extending the continuous monitoring networks and for exploratory studies. Seasonal average or summed ozone concentrations are comparable to the continuous monitoring and, as such, can be used to determine baseline values and longer-term trends. The estimated cost to use the passive samplers is between 10 to 20% of the cost of operating a continuous analyzer. On a regional scale, a combination of passive and active monitors would greatly extend the amount of pollutant information that can be obtained on a limited budget. It may be possible to determine a regional distribution and reference that pattern with a smaller number of sampling sites in future years.

Spatial interpolation of data from multiple monitoring sites is an imperfect tool that can be of assistance in understanding the potential impact regions of air pollutants. Because the interpolations do not take into account such factors at photochemistry, topography, or meteorology, the interpolation estimates are best over extended time periods when these factors would tend to average out. Thus, the areas with the greatest exposure to ozone can be estimated with spatial studies, but not where individual maximum ozone values will occur or where exceedances of a standard are greatest. However, data from an individual monitoring site can be used with a regression model to find an estimate for the seasonal maximum hourly ozone concentration. The technique is not highly accurate and is subject to a number of statistical problems. Taken together, the spatial interpolation gives an idea of where problem areas might be. The concentration magnitudes and regression model estimates give a comparison to a shorter-time standard.

The type of exploratory spatial studies described here can be used for decisions about where to place additional continuous monitors or where vegetation injury studies should be conducted, and as guidance in designing more thorough studies if the need is indicated. Even these limited studies discussed here indicate that the ozone concentrations can vary locally within a rural park when nearby emission sources appear not to be the issue. A comparison of the interpolation estimates from observations with the computer photochemical pollutant model outputs would be interesting as a skill test.

### ACKNOWLEDGMENTS

Much thanks to the resource management staff in the parks that actually did the field work. Special thanks to Chris Holbeck, Donna Meisky, and Katy Warner for helping to plan and execute the detailed field studies in their parks. Miguel Flores has been an inspiration throughout this process. Eva and David Hardison at Research Triangle Institute provided the laboratory skill and patience to make these studies work.
